# Comparative Proteomics of Meat Spoilage Bacteria Predicts Drivers for Their Coexistence on Modified Atmosphere Packaged Meat

**DOI:** 10.3389/fmicb.2020.00209

**Published:** 2020-02-14

**Authors:** Sandra Kolbeck, Christina Ludwig, Chen Meng, Maik Hilgarth, Rudi F. Vogel

**Affiliations:** ^1^Lehrstuhl für Technische Mikrobiologie, Technische Universität München, Freising, Germany; ^2^Bayerisches Zentrum für Biomolekulare Massenspektrometrie, Technische Universität München, Freising, Germany

**Keywords:** meat spoilage, modified atmosphere packaging, comparative proteomics, adaptation, lactic acid bacteria, *Brochothrix thermosphacta*

## Abstract

Besides intrinsic and extrinsic factors such as antagonism for organic substrates or temperature, the storage atmosphere of meat has a high influence on the development of its initial microbiota. Specific modified atmospheres (MAs) selectively suppress growth of aerobic and anaerobic bacteria, thus reshaping the initial microbiota. As some microorganisms are more tolerant to MA, they overgrow competitors and produce metabolites that cause rejection of the product. In order to elucidate responses to different MA by means of metabolic adaptation and competition for organic substrates on meat, the typical representative meat spoilage bacteria *Brochothrix* (*B.*) *thermosphacta* TMW2.2101 and four lactic acid bacteria *Carnobacterium* (*C.*) *divergens* TMW2.1577, *C. maltaromaticum* TMW2.1581, *Leuconostoc* (*L.*) *gelidum* subsp. *gelidum* TMW2.1618 and *L. gelidum* subsp. *gasicomitatum* TMW2.1619 were chosen. Bacteria were grown in sterile glass bottles filled with a meat simulation medium, which was aerated constantly with either air, 100%_N_2_, 30%_CO_2_/70%_O_2_ or 30%_CO_2_/70%_N_2_. Growth of bacteria during incubation at 25°C and stirring at 120 rpm was monitored over 48 h and a label-free quantitative mass spectrometric approach was employed to determine changes within the bacterial proteomes in response to oxygen and carbon dioxide. Both *Leuconostoc* subsp. were intrinsically tolerant to MA, exhibiting no proteomic regulation of enzymes, whereas the other species provide a set of metabolic adaptation mechanism, enabling higher resistance to the detrimental effects of MA. Those mechanisms comprise: enhanced oxidative stress reduction, adjustment of the pyruvate metabolism and catabolic oxygen consumption in response to oxygen and intracellular pH homeostasis, maintenance of osmotic balance and alteration of the fatty acid composition in response to carbon dioxide. We further evaluated the potential of industrial used MA to inhibit specific bacterial spoilage. No bacterial inhibition is predicted for 30%_CO_2_/70%_O_2_ for the analyzed species, whereas 30%_CO_2_/70%_N_2_ predictively inhibits *C. divergens* TMW21577 and *B. thermosphacta* TMW2.2101. Furthermore, species-specific metabolic pathways enabling different and preferential carbon source utilization were identified, which enable non-competitive coexistence of respective bacteria on meat, resulting in synergistic spoilage. In conclusion, this study gives mechanistically explanations of their acknowledged status as typical spoilage organisms on MAP meats.

## Introduction

Food packaging under different modified atmospheres (MAs) has become a common method to counteract deteriorative effects of long time storage of meat, e.g., discoloration and formation of off-odors (Yam et al., 2005; McMillin, 2008). These effects are due to growth of spoilage microorganisms, found as initial contaminations on meat. The initial microbiota is strongly influenced by distribution pathways, processing, slaughtering and storage conditions of the meat (Nychas et al., 2007, 2008). MA selectively suppress members of this initial spoilage microbiota, reshaping it to less sensitive and more tolerant species regarding MA. Those bacteria grow concomitantly upon MAP meat and induce spoilage. They are called ephemeral spoilage organisms (ESOs) as described in previous studies of Nychas et al. (2008) and comprise lactic acid bacteria (LAB), *Brochothrix (B.) thermosphacta*, *Pseudomonas* species, *Enterobacterales* and *Shewanella* and *Aeromonas* species (Lambert et al., 1991; Borch et al., 1996; Ercolini et al., 2006; Nychas et al., 2007; Höll et al., 2016; Hilgarth et al., 2018). As their growth rate and metabolism differs depending on the MA, shelf life can be extended and the sensorial changes can be different (Kakouri and Nychas, 1994; Esmer et al., 2011; Değirmencioğlu et al., 2012).

In order to extend the shelf life, mixtures of the protective gases oxygen and carbon dioxide are applied in modified atmosphere packaged (MAP) meat. Whereas red meat is commonly packaged under a high-oxygen atmosphere containing 30%_CO_2_/70%_O_2_ (Eilert, 2005; Rossaint et al., 2014), white meats, e.g., poultry is packaged with either 30%_CO_2_/70%_N_2_ (McKee, 2007; Sante et al., 2007) or 30%_CO_2_/70%_O_2_ (Eilert, 2005; Rossaint et al., 2014). Oxygen is used to suppress growth of facultative and strict anaerobe bacteria such as *Rahnella aquatilis* or *Clostridia* and further to retain the desirable oxymyoglobin, which is responsible for the red color of meat (Farber, 1991; Lambert et al., 1991; Church, 1994; Ercolini et al., 2006). High oxygen levels induce formation of superoxide (${\text{O}}_{\text{2}}^{-}$) resulting in oxidative stress as described for *Bacteroides thetaiotaomicron* (Pan and Imlay, 2001) and inhibit enzymes containing Fe–S cluster, e.g., dehydratases (Gort and Imlay, 1998), which are present in many strict anaerobic bacteria such as *Clostridia* (Klees et al., 1992; Hofmeister et al., 1994; Scherf et al., 1994). Furthermore, superoxide radicals inhibit other enzymes of the facultatively anaerobe bacterium *Escherichia coli*, which are needed for the catabolism of carbon sources and result in oxidative DNA damage (Farr et al., 1986; Keyer and Imlay, 1996).

The protective gas carbon dioxide has been described as effective against aerobic Gram-negative bacteria, e.g., *Pseudomonas* species (Farber, 1991; Devlieghere and Debevere, 2000). Nevertheless, retarding bacterial growth due to carbon dioxide was also described for Gram-positive bacteria such as *B. thermosphacta* (Molin, 1983; Devlieghere and Debevere, 2000). Despite many investigations on the bacteriostatic action of carbon dioxide, less is known yet about the molecular response to and influence of carbon dioxide on microbial metabolism (Casaburi et al., 2015). Mechanisms of action have been proposed to be an exclusion of oxygen by replacement with carbon dioxide, lowering of intracellular pH by dissociation of formed carbonic acid, an alteration of the structure of the cell membrane and an induction of osmotic unbalance (Sears and Eisenberg, 1961; Daniels et al., 1985). Furthermore, a regulatory effect of single metabolic enzymes has been demonstrated for CO_2_/HCO_3_, e.g., the intracellular level of CO_2_/HCO_3_ induces virulence and toxin production in pathogens such as *Helicobacter pylori* and *Citrobacter rodentium* (Yang et al., 2009; Park et al., 2011).

However, bacterial metabolism is complex, and unknown adaptation mechanisms are hard to uncover and can be overlooked using conventional targeted methods. In contrast, novel “omic”-technologies comprising full genome, transcriptome, metabolome, or proteome analysis enable to capture a non-targeted global “snapshot” of bacterial metabolism. These techniques were recently employed to uncover metabolism, adaptation and interaction of meat spoilage bacteria (Orihuel et al., 2018; Quintieri et al., 2018; Wang et al., 2018; Höll et al., 2019). On this basis, we used a comparative full proteomic analysis, which enabled us to explore global molecular regulation mechanisms of meat spoilage bacteria toward exposure to oxygen and carbon dioxide as well as utilization of distinct organic meat-derived substrates in response to different MA.

## Materials and Methods

### Bacterial Strains

Representative strains for five species isolated from MA packed meat were selected among isolates of previous studies along their abundancy. *B. thermosphacta* TMW 2.2101 was isolated from minced beef (Hilgarth et al., 2019), *Carnobacterium divergens* TMW 2.1577 and *C. maltaromaticum* TMW 2.1581 were isolated from skinless chicken breast (Höll et al., 2016) and *L. gelidum* subsp. *gelidum* TMW 2.1618 and *L. gelidum* subsp. *gasicomitatum* TMW 2.1619 were isolated from beef steaks (Hilgarth et al., 2018).

### Preparation of Precultures

In order to ensure reproducibility, media of all experiments were inoculated from the same respective preculture from glycerol stock cultures prepared in brain heart infusion (BHI) media (Roth, Karlsruhe, Germany). Therefore, bacteria were grown at 25°C aerobically in Erlenmeyer flasks or anaerobically in gas tight Schott bottles. Cultures were harvested, aliquoted, supplemented with 90% glycerol (Gerbu Biotechnik GmbH, Heidelberg, Germany) and frozen at −80°C. Aerobic precultures were used for inoculation of experiments with air and 30%_CO_2_/70%_O_2_ and anaerobic precultures were used for experiments with 100%_N_2_ and 30%_CO_2_/70%_N_2_.

### Experimental Setup

Bacterial cultivation was performed in gas tight locked glass bottles. Glass bottles were filled with 0.4 L MSM media, which was previously employed and described in detail by Kolbeck et al. (2019b). It is specifically adapted to components of real meat mainly consisting of meat extract, glycerol, tween80, and heme. MSM medium was inoculated with an optical density of 0.1 at 590 nm with the previous prepared precultures and stirred at 120 rpm over 48 h. Cultivation temperature was 25 ± 2°C. During cultivation, bottles were constantly aerated with one of the four gas mixtures (air, N_2_, 30%_CO_2_/70%_O_2_, 30%_CO_2_/70%_N_2_). Growth was monitored over 48 h by optical density measurement. Samples for proteomic analysis were taken in exponential growth phase with log CFU ml^–1^ > 7 to comparable to cell counts with those occurring in the relevant time frame during meat spoilage. Bacteria were cultivated in triplicates in separate glass bottles for each gas atmosphere. For proteomic data analysis, samples were taken of each replicate.

### Statistical Data Analysis of the Growth

The values for lag-Phase, maximal optical density (OD_max_) and maximal growth rate (μ_max_) where calculated for each replicate using the open source software RStudio ver. 3.3.0 (RStudio, Inc., Boston, MA, United States) and the CRAN package grofit ver. 1.1.1-1 run with default settings. Significant differences in lag-phases, maximal optical density (OD_max_) and maximal growth rate (μ_max_) were analyzed between the three replicates and the gas atmospheres air, N_2_, 30%_CO_2_/70%_O_2_, 30%_CO_2_/70%_N_2_ for all species by performing a one way analysis of variance (ANOVA), followed by a *post hoc* Tukey test assigning significant differences between means with a confidence interval of 95% (*p* < 0.05).

### Proteomic Sample Preparation

Sample preparation was performed as described in detail previously (Kolbeck et al., 2019a). Briefly, bacterial cells were resuspended in 8M urea lysis buffer and lysed by beads beating. Total protein concentrations were determined using the BCA method. 100 μg of protein extract were reduced with 10 mM DTT and carbamidomethylated with 55 mM chloroacetamide. Subsequently, proteins were digested with 1 μg trypsin overnight at 37°C. Digested peptide samples were desalted and resuspended in 2% acetonitrile, 98% H_2_O, 0.1% formic acid to a final concentration of 0.1 μg/μl.

### LC-MS/MS Data Acquisition

LC-MS/MS measurements were performed on an Ultimate 3000 RSLCnano system coupled to a Q-Exactive HF-X mass spectrometer (Thermo Fisher Scientific). For full proteome analyses 0.5 μg of peptides were delivered to a trap column (ReproSil-pur C18-AQ, 5 μm, Dr. Maisch, 20 mm × 75 μm, self-packed) at a flow rate of 5 μL/min in HPLC grade water with 0.1% formic acid. After 10 min of loading, peptides were transferred to an analytical column (ReproSil Gold C18-AQ, 3 μm, Dr. Maisch, 450 mm × 75 μm, self-packed) and separated using a 50 min gradient from 4 to 32% of solvent B [0.1% formic acid in acetonitrile and 5% (v/v) DMSO) at 300 nL/min flow rate]. Both nanoLC solvents [solvent A = 0.1% formic acid in HPLC grade water and 5% (v/v) DMSO] contained 5% DMSO to boost MS intensity. The Q-Exactive HF-X mass spectrometer was operated in data dependent acquisition (DDA) and positive ionization mode. MS1 spectra (360–1300 m/z) were recorded at a resolution of 60,000 using an automatic gain control (AGC) target value of 3e6 and maximum injection time (maxIT) of 45 ms. Up to 18 peptide precursors were selected for fragmentation in case of the full proteome analyses. Only precursors with charge state 2 to 6 were selected and dynamic exclusion of 25 sec was enabled. Peptide fragmentation was performed using higher energy collision induced dissociation (HCD) and a normalized collision energy (NCE) of 26%. The precursor isolation window width was set to 1.3 m/z. MS2 Resolution was 15.000 with an AGC target value of 1e5 and maximum injection time (maxIT) of 25 ms (full proteome).

### Protein Identification and Quantification by MaxQuant

Peptide identification and quantification was performed using the software MaxQuant (version 1.5.3.30) with its built-in search engine Andromeda (Cox et al., 2011; Tyanova et al., 2016a). MS2 spectra were searched against the NCBI proteome databased of *L. gelidum* subsp. *gelidum* TMW2.1618 (CP017196), *L. gelidum* subsp. *gasicomitatum* TMW2.1619 (CP017197), *C. divergens* TMW2.1577 (RSDV00000000.1), *C. maltaromaticum* TMW2.1581 (CP016844) and *B. thermosphacta* TMW2.2101 (RSDU00000000.1), supplemented with common contaminants (built-in option in MaxQuant). Trypsin/P was specified as proteolytic enzyme. Precursor tolerance was set to 4.5 ppm, and fragment ion tolerance to 20 ppm. Results were adjusted to 1% false discovery rate (FDR) on peptide spectrum match (PSM) level and protein level employing a target-decoy approach using reversed protein sequences. The minimal peptide length was defined as seven amino acids, the “match-between-run” function was disabled. For full proteome analyses carbamidomethylated cysteine was set as fixed modification and oxidation of methionine and N-terminal protein acetylation as variable modifications.

### Statistical Data Analysis by Perseus

Either labeled free quantification (LFQ) values were used from MaxQuant for identifying significantly different regulated proteins between two sampling conditions or intensity based absolute quantification (iBAQ) values to compare general expression of proteins within each sample. LFQ and iBAQ values were identically possessed using the open source software Perseus (Tyanova et al., 2016b). Statistical data analyzation was done applying the following pipeline: (I) Data clean-up by removing proteins from potential contaminants, only identified by site or reverse, (II) log_2_ data transformation and normalization, (III) removing proteins only identified in one of three replicates, (IV) calculating the mean of three replicates, (V) performing a Welch *t*-test of two sampling conditions (only with LFQ values), (VI) exporting data to excel (Supplementary Table S1). Afterward, proteins were filtered based on the following parameters: *q*-value < 0.05 and log_2_ fold change > = 2. The total number of proteins significantly differentially (*q*-value < 0.05 and log_2_ fold change > 2) up or down regulated for each species and each comparison are to be seen in Supplementary Table S2.

### Data Interpretation

**FIGURE 1:**
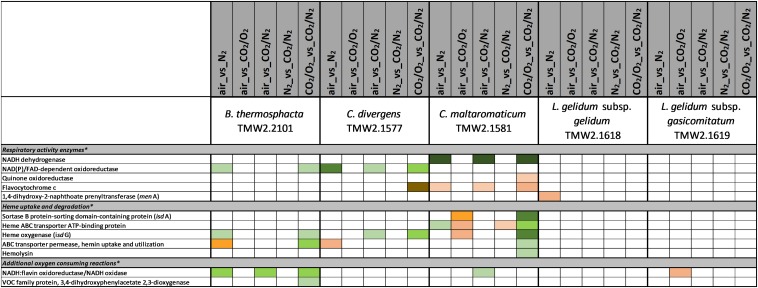
Proteomic analysis of enzymes involved in respiratory activity of five meat spoilage bacteria grown under different gas atmospheres. Data are based on three independent replicates. Proteins were classified as significantly regulated with *p* < 0.05 (Welch *t*-test). Colors indicate the effect of regulation with 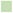 log_2_(ddiff.) = 2, 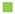 log_2_ (diff.) = 3, 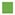 log_2_ (diff.) = 4, 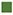 log_2_ (diff.) = 5, 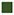 log_2_ (diff.) = 6 and 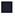 log_2_ (diff.) = 7 for upregulated proteins and 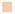 log_2_ (diff.) = 2, 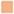 log_2_ (diff.) = 3, 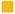 log_2_ (diff.) = 4, 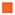 log_2_ (diff.) = 5, 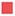 log_2_ (diff.) = 6, 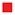 log_2_ (diff.) = 7, 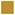 log_2_ (diff.) = 8 and 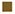 log_2_ (diff.) = 9 for downregulated proteins. ^∗^ Functional categories are based on NCBI annotation, TIGR annotation, SEED annotation, KEGG annotation, and own research.

**FIGURE 2:**
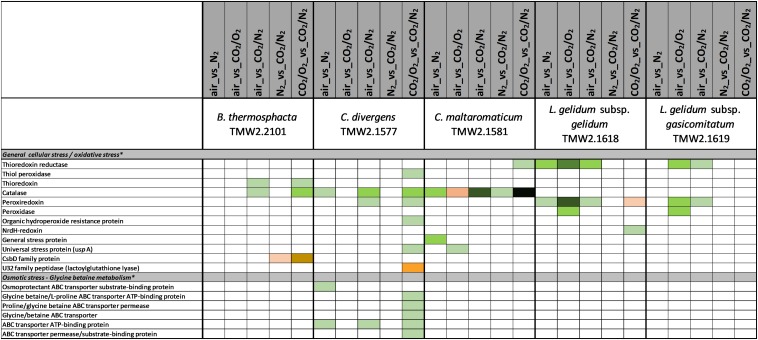
Proteomic analysis of enzymes involved in cell stress metabolism of five meat spoilage bacteria grown under different gas atmospheres. Data are based on three independent replicates. Proteins were classified as significantly regulated with *p* < 0.05 (Welch *t*-test). Colors indicate the effect of regulation with 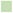 log_2_(diff.) = 2, 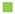 log_2_ (diff.) = 3, 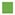 log_2_ (diff.) = 4, 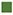 log_2_ (diff.) = 5, 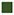 log_2_ (diff.) = 6 and 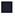 log_2_ (diff.) = 7 for upregulated proteins and 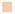 log_2_ (diff.) = 2, 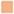 log_2_ (diff.) = 3, 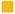 log_2_ (diff.) = 4, 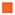 log_2_ (diff.) = 5, 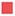 log_2_ (diff.) = 6, 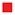 log_2_ (diff.) = 7, 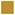 log_2_ (diff.) = 8 and 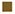 log_2_ (diff.) = 9 for downregulated proteins. ^∗^ Functional categories are based on NCBI annotation, TIGR annotation, SEED annotation, KEGG annotation, and own research.

**FIGURE 3:**
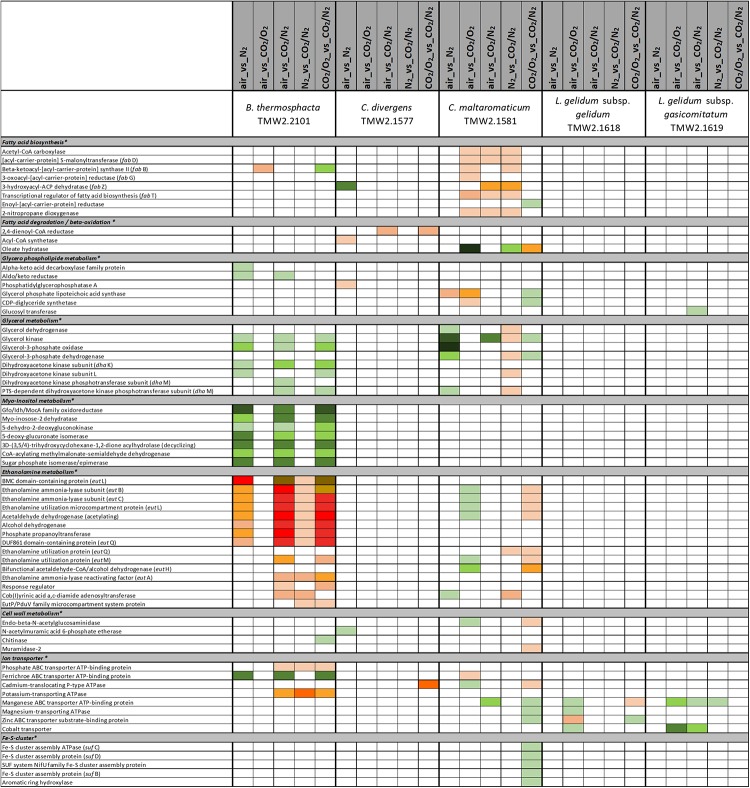
Proteomic analysis of enzymes involved in cell membrane and cell wall metabolism of five meat spoilage bacteria grown under different gas atmospheres. Data are based on three independent replicates. Proteins were classified as significantly regulated with *p* < 0.05 (Welch *t*-test). Colors indicate the effect of regulation with 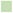 log_2_(diff.) = 2, 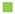 log_2_ (diff.) = 3, 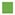 log_2_ (diff.) = 4, 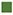 log_2_ (diff.) = 5, 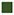 log_2_ (diff.) = 6 and 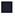 log_2_ (diff.) = 7 for upregulated proteins and 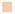 log_2_ (diff.) = 2, 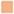 log_2_ (diff.) = 3, 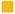 log_2_ (diff.) = 4, 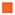 log_2_ (diff.) = 5, 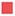 log_2_ (diff.) = 6, 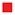 log_2_ (diff.) = 7, 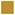 log_2_ (diff.) = 8 and 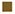 log_2_ (diff.) = 9 for downregulated proteins. ^∗^ Functional categories are based on NCBI annotation, TIGR annotation, SEED annotation, KEGG annotation, and own research.

**FIGURE 4:**
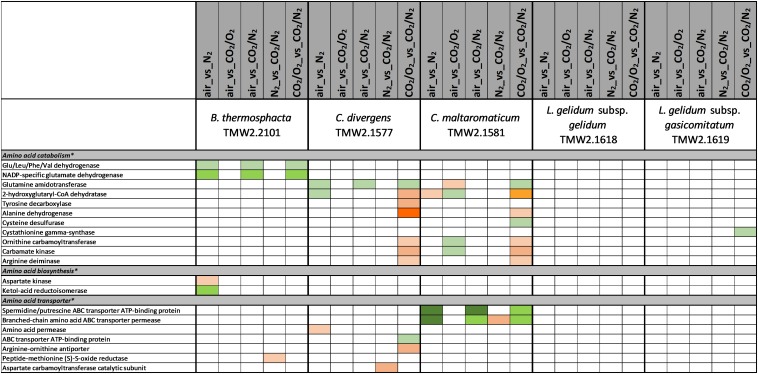
Proteomic analysis of enzymes involved in amino acid metabolism of five meat spoilage bacteria grown under different gas atmospheres. Data are based on three independent replicates. Proteins were classified as significantly regulated with *p* < 0.05 (Welch *t*-test). Colors indicate the effect of regulation with 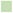 log_2_(diff.) = 2, 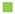 log_2_ (diff.) = 3, 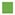 log_2_ (diff.) = 4, 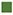 log_2_ (diff.) = 5, 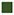 log_2_ (diff.) = 6 and 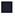 log_2_ (diff.) = 7 for upregulated proteins and 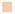 log_2_ (diff.) = 2, 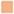 log_2_ (diff.) = 3, 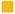 log_2_ (diff.) = 4, 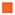 log_2_ (diff.) = 5, 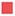 log_2_ (diff.) = 6, 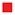 log_2_ (diff.) = 7, 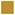 log_2_ (diff.) = 8 and 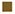 log_2_ (diff.) = 9 for downregulated proteins. ^∗^ Functional categories are based on NCBI annotation, TIGR annotation, SEED annotation, KEGG annotation, and own research.

**FIGURE 5:**
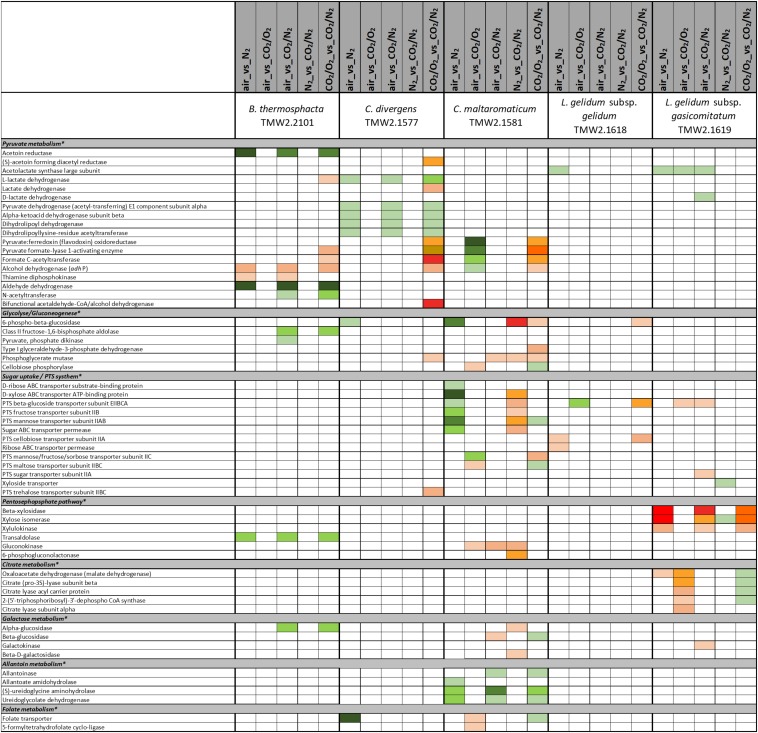
Proteomic analysis of enzymes involved in carbohydrate metabolism of five meat spoilage bacteria grown under different gas atmospheres. Data are based on three independent replicates. Proteins were classified as significantly regulated with *p* < 0.05 (Welch *t*-test). Colors indicate the effect of regulation with 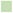 log_2_(diff.) = 2, 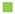 log_2_ (diff.) = 3, 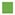 log_2_ (diff.) = 4, 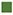 log_2_ (diff.) = 5, 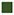 log_2_ (diff.) = 6 and 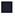 log_2_ (diff.) = 7 for upregulated proteins and 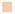 log_2_ (diff.) = 2, 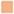 log_2_ (diff.) = 3, 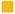 log_2_ (diff.) = 4, 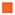 log_2_ (diff.) = 5, 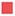 log_2_ (diff.) = 6, 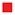 log_2_ (diff.) = 7, 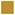 log_2_ (diff.) = 8 and 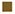 log_2_ (diff.) = 9 for downregulated proteins. ^∗^ Functional categories are based on NCBI annotation, TIGR annotation, SEED annotation, KEGG annotation, and own research.

**FIGURE 6:**
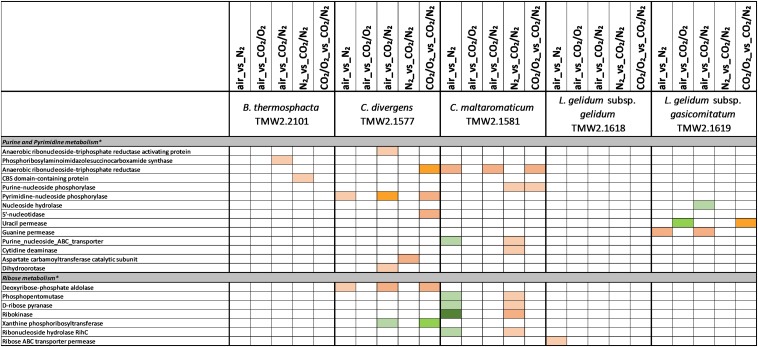
Proteomic analysis of enzymes involved in purine/pyrimidine and ribose metabolism of five meat spoilage bacteria grown under different gas atmospheres. Data are based on three independent replicates. Proteins were classified as significantly regulated with *p* < 0.05 (Welch *t*-test). Colors indicate the effect of regulation with 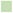 log_2_(diff.) = 2, 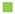 log_2_ (diff.) = 3, 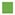 log_2_ (diff.) = 4, 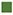 log_2_ (diff.) = 5, 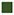 log_2_ (diff.) = 6 and 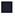 log_2_ (diff.) = 7 for upregulated proteins and 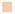 log_2_ (diff.) = 2, 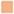 log_2_ (diff.) = 3, 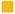 log_2_ (diff.) = 4, 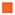 log_2_ (diff.) = 5, 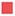 log_2_ (diff.) = 6, 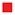 log_2_ (diff.) = 7, 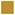 log_2_ (diff.) = 8 and 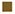 log_2_ (diff.) = 9 for downregulated proteins. ^∗^ Functional categories are based on NCBI annotation, TIGR annotation, SEED annotation, KEGG annotation, and own research.

Figures 1–6 include proteins from Supplementary Table S2, which could be assigned to a specific metabolism (respiratory chain metabolism Figure 1, cell stress metabolism Figure 2, cell membrane and cell wall metabolism Figure 3, amino acid metabolism Figure 4, carbohydrate metabolism Figure 5 and purine/pyrimidine and ribose metabolism Figure 6). Assignment of proteins to a specific metabolic pathway was done using functional annotations provided by the databases NCBI (Localization), RAST (Category, Subcategory, Subsystem, Role), TIGR (ROLEmain, ROLEsub1, seqdesc) and KEGG (GO-number). Additionally, manual curation employing additional BLAST search was performed to ensure correct annotation and assignment of proteins to metabolic pathways.

Five different approaches were performed on proteins shown in Figures 1–6, to identify the effects of oxygen (21%) (Approach 1), high oxygen (70%) (Approach 2) and carbon dioxide under oxic (Approaches 3 and 5) and anoxic conditions (Approaches 4 and 5), on the proteome of bacteria (Table 1). The effect of oxygen (21%) can be seen by focusing on the three comparisons air_vs_N_2_, air_vs_CO_2_/N_2_ and CO_2_/O_2__vs_CO_2_/N_2_ (Approach 1). The effect of high oxygen concentrations (70%) can be revealed looking at the second-tier comparison of proteins differentially regulated between the two comparisons air_vs_N_2_ and CO_2_/O_2__vs_CO_2_/N_2_ as well as looking at the comparison air_vs_CO_2_/O_2_ (Approach 2). The effect of carbon dioxide under oxic conditions can be seen comparing the expression of proteins detected under air_vs_CO_2_/O_2_ atmosphere (Approach 3). The effect of carbon dioxide under anoxic conditions can be seen from the comparison N_2__vs_CO_2_/N_2_ (Approach 4). Furthermore, a second-tier comparison of proteins differentially regulated between the two comparisons air_vs_N_2_ and CO_2_/O_2__vs_CO_2_/N_2_, also revealed the effect of carbon dioxide in response to absence or presence of oxygen (Synergistic effect, Approach 5).

**TABLE 1:** Evaluation method to identify the effect of the protective gases carbon dioxide and oxygen on the metabolism of meat spoilage bacteria.

**Approach**	**Read out**	**Comparison**
1	Effect of oxygen (oxic to anoxic conditions)	Air vs. N_2_ Air vs. 30%_CO_2_/ 70%_N_2_ 30%_CO_2_/70%_O_2_ vs. 30%_CO_2_/70%_N_2_
2	Effect of high oxygen concentration (70%) compared to low oxygen concentration (21%)	(Air vs. N_2_) vs. (30%_CO_2_/70%_O_2_ vs. 30%_CO_2_/70%_N_2_) Air vs. 30%_CO_2_/70%_O_2_
3	Effect of carbon dioxide under oxic conditions	Air vs. 30%_CO_2_/70%_O_2_
4	Effect of carbon dioxide under anoxic conditions	N_2_ vs. 30%_CO_2_/70%_N_2_
5	Effect of carbon dioxide under oxic compared to anoxic conditions	(Air vs. N_2_) vs. (30%_CO_2_/70%_O_2_ vs. 30%_CO_2_/70%_N_2_)

*Analysis are based on proteomic data.*

## Results

### Growth of Meat Spoilage Bacteria Under Different Protective Gas Atmospheres

Growth of bacteria was detected for all species under different protective gas atmospheres. Table 2 summarizes the main growth parameters OD_max_, lag-phase and μ_max_ for the bacterial species. Details are provided in Supplementary Figure S1.

**TABLE 2:** Growth of the meat spoilage bacteria *B. thermosphacta* TMW2.2101, *C. divergens* TMW2.1577, *C. maltaromaticum* TMW2.1581, *L. gelidum* subsp. *gelidum* TMW2.1618, and *L. gelidum* subsp. *gasicomitatum* TMW2.1619 under different protective gas atmospheres in meat simulation media.

***B. thermosphacta* TMW2.2101**	**Lag-phase [h] ^A, C–F^**	***SE* [h]**	**OD_max_**	***SE***	**μ_max_ [division/h] ^E, F^**	***SE* [division/h]**
Air	17.78	1.31	2.06	0.59	0.15	0.03
N_2_	0	0	0.96	0.08	0.08	0.00
CO_2_/N_2_	15.47	0.35	0.56	0.01	0.02	0.00
CO_2_/O_2_	38.71	0.55	4.50	1.68	0.47	0.15
***C. divergens* TMW2.1577**	**Lag-phase [h] ^A–F^**		**OD_max_^A,B,E,F^**		**μ_max_ [division/h] ^B, F^**	
Air	17.64	1.07	1.30	0.21	0.08	0.02
N_2_	0	0.17	0.58	0.00	0.05	0.00
CO_2_/N_2_	9.65	2.4	0.56	0.00	0.03	0.00
CO_2_/O_2_	28.57	1.38	1.40	0.06	0.07	0.00
***C. maltaromaticum* TMW2.1581**	**Lag-phase [h] ^A–D, F^**		**OD_max_^A, C, E, F^**		**μ_max_ [division/h] ^C, E, F^**	
Air	21.16	0.79	2.44	0.24	0.09	0.01
N_2_	5.46	0.06	1.44	0.02	0.07	0.00
CO_2_/N_2_	2.06	0.39	2.12	0.02	0.09	0.00
CO_2_/O_2_	7.33	0.16	4.00	0.31	0.20	0.01
***L. gelidum* subsp. *gelidum* TMW2.1618**	**Lag-phase [h] ^B–E^**		**OD_max_^A–C, E, F^**		**μ_max_ [division/h] ^B, C, E, F^**	
Air	7.40	0.74	0.84	0.02	0.04	0.00
N_2_	8.50	0.78	0.75	0.01	0.03	0.00
CO_2_/N_2_	3.31	0.60	0.73	0.00	0.03	0.00
CO_2_/O_2_	3.31	0.20	1.20	0.01	0.06	0.00
***L. gelidum* subsp. *gasicomitatum* TMW2.1619**	**Lag-phase [h] ^C^**		**OD_max_^A–F^**		**μ_max_ [division/h] ^B–F^**	
Air	7.23	0.66	0.89	0.02	0.04	0.00
N_2_	8.30	0.53	0.72	0.01	0.04	0.00
CO_2_/N_2_	9.21	0.34	1.51	0.01	0.07	0.00
CO_2_/O_2_	9.91	0.21	1.03	0.02	0.06	0.00

*All numbers are average values based on three independent replicates. Superscript letters indicate significant differences (confidence interval 95%; *p* < 0.05) between calculated parameters comparing the cultivation conditions air vs. N_2_ (A), air vs. 30%_CO_2_/70%_N_2_ (B), air vs. 30%_CO_2_/70%_O_2_ (C), N_2_ vs. 30%_CO_2_/70%_N_2_ (D), N_2_ vs. 30%_CO_2_/70%_O_2_ (E) and 30%_CO_2_/70%_N_2_ vs. 30%_CO_2_/70%_O_2_ (F).*

Significant enhanced growth could be detected for *C. maltaromaticum* TMW2.1581 and *Leuconostoc gelidum* subsp. *gelidum* TMW2.1618 by comparing the atmospheres air vs. 30%_CO_2_/70%_O_2_. The other species did not show enhanced growth. Furthermore, *C. divergens* TMW2.1577 and *Leuconostoc gelidum* subsp. *gasicomitatum* TMW2.1619 were the only species exhibiting enhanced growth by comparing the cultivation conditions air vs. 30%_CO_2_/70%_N_2_. A significant prolongation of the lag phase was observed for *B. thermosphacta* TMW2.2101 and *C. divergens* TMW2.1577 in presence of carbon dioxide, comparing N_2_ vs. 30%_CO_2_/70%_N_2_ and air vs. 30%_CO_2_/70%_O_2_. No prolongation of the lag phase was observed for the other three species in response to carbon dioxide.

### Proteomic Analysis of Metabolic Pathways of Different Meat Spoilage Bacteria

A label-free quantitative proteomic approach was applied to identify differentially regulated proteins of meat spoilage bacteria cultivated under protective gas atmospheres (Supplementary Table S2). In total, 74% (1696 of 2285) of all encoded proteins of *B. thermosphacta* TMW2.2101, 73% (1811 of 2490) of *C. divergens* TMW2.1577, 67% (2152 of 3205) of *C. maltaromaticum* TMW2.1581, 86% (1375 of 1605) of *L. gelidum* subsp. *gelidum* TMW2.1618 and 82% (1420 of 1740) of *L. gelidum* subsp. *gasicomitatum* TMW2.1619 were detected as expressed and quantified by MaxQuant. After statistical data analysis by Perseus, up to 47 proteins remained as differential expressed in at least one comparison for *B. thermosphacta* TWM2.2101, 41 for *C. divergens* TMW2.1577, 46 for *C. maltaromaticum* TMW2.1581, 14 for *L. gelidum* subsp. *gelidum* TMW2.1618 and 26 for *L. gelidum* subsp. *gasicomitatum* TMW2.1619.

Five different approaches were performed on these significantly regulated proteins, to identify the effects of oxygen (21%) (Approach 1), high oxygen (70%) (Approach 2) and carbon dioxide under oxic (Approaches 3 and 5) and anoxic conditions (Approaches 4 and 5), on the proteome of bacteria (Table 1). The readout of this approaches is listed in Table 3 and explained in detail in the following sections. An overall summary of the inhibitory effect of carbon dioxide and MA on bacteria is given in Table 4.

**TABLE 3:** Regulatory effect of the protective gas atmospheres carbon dioxide and oxygen on metabolic pathways of the meat spoilage bacteria *B. thermosphacta* TMW2.2101, *C. divergens* TMW2.1577, *C. maltaromaticum* TMW2.1581, *L. gelidum* subsp. *gelidum* TMW2.1618, and *L. gelidum* subsp. *gasicomitatum* TMW2.1619.

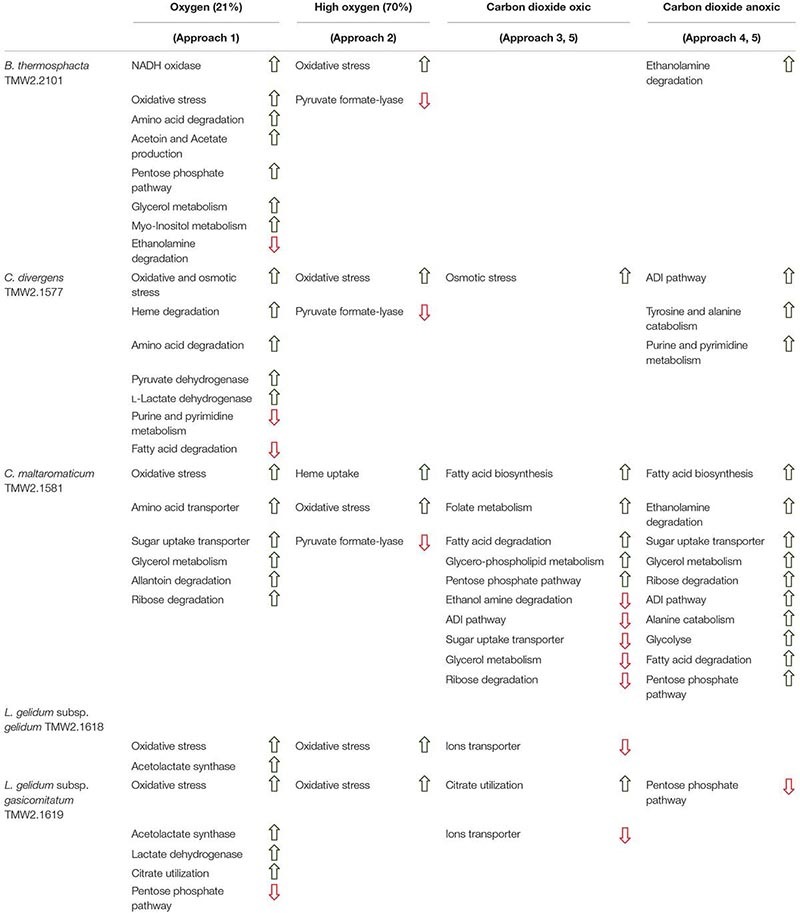

*Arrows indicate if a metabolic pathway is upregulated or downregulated in the corresponding gas atmosphere.*

**TABLE 4:** Summary of the effect of carbon dioxide on the lag-phase, maximal optical density OD_max_, maximal growth rate μ_max_ and the proteome (− no effect, + moderate effect, ++ strong effect).

	**Effect of CO_2_ on**			**CO_2_**	**Overall inhibition on**	
	**Lag-Phase**	**μ_max_ and OD_max_**	**Proteome**	**Inhibition and adaptation**	**White meat (30%_CO_2_/70%_N_2_)**	**Red meat (30%_CO_2_/70%_O_2_)**
*B. thermosphacta* TMW2.2101	+	−	+	Inhibition but adaptation	Yes	No
*C. divergens* TMW2.1577	+	−	+ +	Inhibition but adaptation	Yes	No
*C. maltaromaticum* TMW2.1581	−	−	+ +	No inhibition due to adaptation	No	No
*L. gelidum* subsp. *gelidum* TMW21618	−	−	−	No inhibition/adaptation	No	No
*L. gelidum* subsp. *gasicomitatum* TMW2.1619	−	−	−	No inhibition/adaptation	No	No

*Classification in “moderate” and “strong” was graduated by taking into account the number of proteins differentially regulated comparing gas atmospheres with and without carbon dioxide. “Inhibition” is defined based on a prolongation of the lag phase whereas “adaptation” is supposed when bacteria exhibit a proteomic regulation as well as no effect on μ_*max*_ and OD_*max*_ due to carbon dioxide. Overall conclusion about predicted effective inhibition of meat-spoiling bacteria by the protective gases oxygen and carbon dioxide as used in modified atmospheres of white (30%_CO_2_/70%_N_2_) and red (30%_CO_2_/70%_O_2_) meat packages.*

### Metabolic Response to Aerobic Conditions (Approach 1)

Several metabolic pathways of bacteria were affected when exposed to aerobic conditions containing 21% oxygen. Highest metabolic response was detected for *B. thermosphacta* TMW2.2101 exhibiting eight pathways being regulated followed by *C. divergens* TMW2.1577 (7 pathways), *C. maltaromaticum* TMW2.1581 (6 pathways) and both *L. gelidum* subspecies (2 and 5 pathways). Regulated pathways comprised organic substrate utilization, e.g., amino acid, glycerol, purine and pyrimidine and ribose degradation as well as sugar uptake. Beside this, all bacteria shared an upregulation of oxidative stress enzyme as common metabolic response to oxic conditions. Furthermore single oxygen consuming enzymes, e.g., acetolactate synthase or NADH oxidase were upregulated for *L. gelidum* subspecies or *B. thermosphacta* TMW2.2101.

### Metabolic Response to 70% Oxygen (Approach 2)

Less metabolic pathways were regulated when bacteria were exposed to high oxygen conditions (70% oxygen) compared to normal aerobe conditions (21% oxygen). In total, up to three metabolic pathways were regulated for each species. Shared metabolic pathways comprised an upregulation of oxidative stress enzymes. *B. thermosphacta* TMW2.2101 and both *Carnobacteria* species exhibited a downregulation of the pyruvate formate-lyse in response to high oxygen concentrations.

### Metabolic Response to Carbon Dioxide Under Oxic Conditions (Approaches 3 and 5)

Various differences between species were observed concerning their metabolic response to carbon dioxide under oxic conditions. No proteomic regulation of pathways was observed for *B. thermosphacta* TMW2.2101, a bit more for *C. divergens* TMW2.1577 and both *L. gelidum* subspecies (up to two pathways) and a high proteomic regulation was observed for *C. maltaromaticum* TMW2.1581 (10 pathways). Proteins influencing intracellular osmolarity, e.g., osmotic stress proteins and ion transporters were significantly regulated for *C. divergens* TWM2.1577 and both *L. gelidum* subspecies. *C. maltaromaticum* TMW2.1581 exhibited an upregulation of enzymes involved in fatty acid biosynthesis and degradation as well as a downregulation of enzymes involved in sugar metabolism, adenine and ethanolamine degradation.

### Metabolic Response to Carbon Dioxide Under Anoxic Conditions (Approaches 4 and 5)

Metabolic response to carbon dioxide under anoxic conditions was not detected for *L. gelidum* subsp. *gelidum* TMW2.1618. *C. maltaromaticum* TMW2.1581 showed again highest metabolic regulation, whereas the other three species showed little metabolic regulation to carbon dioxide. Both *Carnobacteria* upregulated the ADI pathway and specific enzymes for amino acid degradation in response to carbon dioxide. Additionally, *C. maltaromaticum* TMW2.1581 upregulated several fatty acid biosynthesis and degradation enzymes as well as enzymes for ethanolamine degradation. Latter was also upregulated by *B. thermosphacta* TMW2.2101 in response to carbon dioxide.

### Proteomic Regulation of Respiratory Chain Enzymes

Furthermore, general non-regulated protein expression of respiratory chain enzymes was separately analyzed in response to different gas atmospheres using iBAQ values (Supplementary Table S1). All enzymes needed to establish a functional respiratory chain were expressed for all bacteria independently of the gas atmosphere.

## Discussion

Protective gas atmospheres comprising oxygen and carbon dioxide are commonly used to selectively inhibit microbial growth on meat (Devlieghere and Debevere, 2000; McMillin, 2008; Esmer et al., 2011). In the past, studies have aimed to reveal the effect of different gas atmospheres on the spoilage of meat, by analyzing production of metabolites, e.g., acetate, ethanol, diacetyl, 2.3-butanediol, or volatile components (Skandamis and Nychas, 2002; Balamatsia et al., 2006, 2007; Ercolini et al., 2006; Casaburi et al., 2015). Those studies focused on spoilage induced by a whole microbial consortium. Nowadays, novel methods enable a more detailed insight into the *in situ* metabolism of individual meat spoilage bacteria, e.g., using a meta-transcriptomic approach as employed by Höll et al. (2019). In this study, a quantitative proteomic approach was applied to provide mechanistic insight in regulation mechanisms of individual meat spoilage bacteria to oxygen and carbon dioxide upon different gas atmosphere compositions. This approach demonstrated that individual species use different adaptation mechanisms and encode for specific metabolic pathways to cope with MAs used in meat preservation and bypass competition for nutrients by preferential use of distinct organic substrates which can be found on meat.

### Effect of Different Gas Atmospheres on the Protein Expression of Respiratory Chain Enzymes

Consumption of intracellular oxygen by respiration can be considered as one major adaptation mechanism of bacteria upon high oxygen concentrations. This has been proven for the analyzed strains in a previous study that aimed to quantify oxygen consumption (Kolbeck et al., 2019b). As almost no significant differential expression of respiratory enzymes was detected in this study, we conclude that respiratory enzymes are constitutively expressed for the analyzed LABs and *B. thermosphacta*, enabling bacteria to immediately respond to oxygen. This is in congruent with the findings of other studies, dealing with the expression of respiratory chain enzymes of LABs (Pedersen et al., 2012).

### Effect of Oxygen and Carbon Dioxide on the Metabolism of *B. thermosphacta* TMW 2.2101

We detected several adaptation mechanism of *B. thermosphacta* TMW2.2101 to the presence of oxygen, including oxygen consumption by the enzyme NADH oxidase, a downregulation of the oxygen sensitive pyruvate formate-lyase and a reduction of oxidative stress by the enzymes thioredoxin and catalase. This is in accordance to a previous study, where a high oxygen uptake rate and resistance to hydrogen peroxide was measured for the same strain of *B. thermosphacta* (Kolbeck et al., 2019b). Regarding an adaptation mechanism to carbon dioxide, we detected an enhanced expression of enzymes needed to degrade ethanolamine under anoxic conditions. Ethanolamine is abundant in mammalian cell membranes and can be found as part of the head group of phospholipids (Garsin, 2010). Degradation of the meat derived metabolite ethanolamine results in the production of ammonia and acetyl-CoA. As an intracellular pH-reduction is known to be caused by carbonic acid from carbon dioxide and its dissociation into carbonate and protons (Daniels et al., 1985; Smith et al., 1990), we suggest ammonia production by ethanolamine uptake and degradation as one adaptation mechanism of *B. thermosphacta* TMW2.2101 to carbon dioxide, which enables intracellular pH homeostasis. We further detected a prolonged lag phase for this bacterium in response to carbon dioxide exposure. As maximum OD and growth rate were not significantly different comparing air vs. 30%_CO_2_/70%_O_2_, we conclude that the inhibitory effect of carbon dioxide in 30%_CO_2_/70%_O_2_ MAP meat for *B. thermosphacta* TMW2.2101 is foiled by the presence of high oxygen concentrations. Nevertheless, the combination of 30% carbon dioxide and absence of oxygen, needed for respiration, can effectively inhibit growth of *B. thermosphacta* in white meat packages as previously demonstrated by Höll et al. (2016).

### Effect of Oxygen and Carbon Dioxide on the Metabolism of *C. divergens* TMW 2.1577

*Carnobacterium divergens* is one of the dominating spoilage microorganisms found on high oxygen packaged meat (Nieminen et al., 2011; Doulgeraki et al., 2012; Höll et al., 2016). As identified in this study, mechanisms of *C. divergens* TMW2.1577 to withstand the effect of high oxygen concentrations comprise an enhanced protein expression for reduction of oxidative stress and regulation of the pyruvate metabolism by downregulating the oxygen sensitive pyruvate formate-lyase. Adaptation mechanisms of *C. divergens* TMW2.1577 to carbon dioxide under oxic conditions comprise maintenance of the osmotic balance by upregulating several glycine betaine transporters. A disturbance of the cell membrane permeability to ionic species due to carbon dioxide has previously been described by Sears and Eisenberg (1961). Thus, by uptaking glycine betaine, present on chicken (Jung et al., 2015) and beef (Zeisel et al., 2003), bacteria are able to compensate changes in environmental osmolarity, as described by Csonka (1989). To compensate intracellular pH reduction by carbon dioxide, *C. divergens* TMW2.1577 increased the production of basic metabolites, e.g., ammonia and biogenic amines by upregulating the corresponding enzymes alanine dehydrogenase, tyrosine decarboxylase, and enzymes of the ADI pathway under anoxic conditions. Similar to *B. thermosphacta* TMW2.2101, *C. divergens* TMW2.1577 showed prolonged lag-phases in response to carbon dioxide but exhibited no significant difference in its total growth (OD_max_, μ_max_) comparing air vs. 30%_CO_2_/70%_O_2_. Thus, we conclude that *C. divergens* TMW2.1577 is sensitive to carbon dioxide but exhibits fast proteomic adaptation prior to the lag phase and further favors high oxygen concentrations resulting in no effective inhibition by 30%_CO_2_/70%_O_2_ gas atmosphere as used in red meat. Nevertheless, a significantly lower max OD was observed by comparing the gas mixture used in white meat (30%_CO_2_/70%_N_2_) vs. air, thus assuming effective inhibition of *C. divergens* TMW2.1577 in white meat packages, which might not result from carbon dioxide rather than the absence of growth promoting oxygen.

### Effect of Oxygen and Carbon Dioxide on the Metabolism of *C. maltaromaticum* TMW 2.1581

*C. maltaromaticum* TMW2.1581 exhibited the strongest regulation mechanism on proteome level, i.e., the highest number of differentially expressed proteins compared to other species. Besides reduction of oxidative stress and regulation of the pyruvate metabolism, we also predict oxygen consumption by overexpression of heme uptake system for enhanced respiratory chain activity as mechanisms of *C. maltaromaticum* TMW2.1581 to high oxygen concentrations. Regarding the influence of carbon dioxide, we detected an antidromic regulation of metabolic pathways of *C. maltaromaticum* TMW2.1581 under oxic compared to anoxic conditions, indicating its regulatory machinery being able to easily cope to a changing environment. In accordance to *C. divergens* TMW2.1577 and *B. thermosphacta* TMW2.2101, adaptation mechanism of *C. maltaromaticum* TMW2.1581 to carbon dioxide comprise an upregulation of enzymes of the ethanolamine degradation pathway, ADI pathway and the alanine dehydrogenase enzyme under anoxic conditions. Additionally, changes of the cell membrane composition appear to be one major adaptation mechanism of *C. maltaromaticum* TWM2.1577 to the presence of carbon dioxide. This could be seen by an upregulation of enzymes of the fatty acid biosynthesis cluster under anoxic conditions. Previous studies dealing with interfacial tension measurements also assumed that the cell membrane is one major site of action of carbon dioxide (Sears and Eisenberg, 1961; Daniels et al., 1985). In conclusion, *C. maltaromaticum* TMW2.1581 showed distinct proteomic adaptation mechanism to carbon dioxide and exhibited no inhibition in its growth comparing air vs. 30%_CO_2_/70%_N_2_ or air vs. 30%_CO_2_/70%_O_2_. Taken together, we conclude that none of the two protective gas atmospheres used in MAP red and white meat effectively inhibit *C. maltaromaticum* TMW2.1581 and thus do not prevent meat spoilage by this strain.

### Effect of Oxygen and Carbon Dioxide on the Metabolism of *L. gelidum* subsp. *gelidum* TMW2.1618

Less proteomic regulation in response to oxygen and carbon dioxide was detected for *L. gelidum* subsp. *gelidum* TMW2.1618 compared to the other species. Besides respiration, oxygen consumption by the activity of the enzyme acetolactate synthase has to be considered as one major adaptation to oxygen of *L. gelidum* subsp. *gelidum* TMW2.1618. Furthermore, *L. gelidum* subsp. *gelidum* TMW2.1618 reduces oxidative stress by an enhanced expression of the enzymes thioredoxin reductase and peroxiredoxin. No metabolic response to carbon dioxide was detected for *L. gelidum* subsp. *gelidum* TMW2.1618, except several ion transporters being downregulated under oxic conditions. This might be due to the altered ion permeability of the cell membrane caused by carbon dioxide as mentioned above (Sears and Eisenberg, 1961). Similar to *C. maltaromaticum*, *L. gelidum* subsp. *gelidum* TMW2.1618 showed no inhibition comparing air vs. 30%_CO_2_/70%_N_2_ or air vs. 30%_CO_2_/70%_O_2_, indicating that growth and metabolism of this bacterium is not effected by CO_2_ (30%) at all. As even a significant higher growth was detected for this bacterium comparing air vs. 30%_CO_2_/70%_O_2_, we conclude, that the typical atmospheres used in white and red meat packages do not effectively inhibit growth of *L. gelidum* subsp. *gelidum* as demonstrated for the representative strain TMW2.1618.

### Effect of Oxygen and Carbon Dioxide on the Metabolism of *L. gelidum* subsp. *gasicomitatum* TMW 2.1619

*Leuconostoc gelidum* subsp. *gasicomitatum* TMW2.1619 exhibited highly similar adaptation mechanism to oxygen and carbon dioxide as described for *L. gelidum* subsp. *gelidum* TMW2.1618. We also measured no inhibition on the growth of *L. gelidum* subsp. *gasicomitatum* TMW2.1619 due to carbon dioxide. Furthermore, a significantly higher growth was detected comparing gas atmospheres used in white meat packaging (30%_CO_2_/70%_N_2_) compared to air. Thus, we also predict no inhibition or even promoted growth of *L. gelidum* subsp. *gasicomitatum* TMW2.1619 in MAP white or red meat due to 30% CO_2_ or high oxygen concentrations, respectively.

### Adaptation to Different Carbon Sources of Meat-Spoiling Bacteria Under Oxic and Anoxic Conditions

There is a high diversity of different nutrients on red and white meats, which makes it unlikely that members of one species could completely occupy such a habitat outcompeting any others. This is indicated by the finding of typical consortia. Adaptation to the utilization of different carbon sources appears essential for the coexistence and microbiome dynamics of meat bacteria during product storage. In the chosen models, all bacteria utilize glucose and ribose as basic carbon sources, as could be predicted by a constitutive expression of the corresponding metabolic enzymes independent of the gas atmosphere. Beside this basic carbon sources, individual adaptation to specific other carbon sources was detected for each species. These should be even more decisive in meat spoilage as sugars are readily depleted in the initial spoilage phase employing a diverse microbiota. Regarding *B. thermosphacta* TMW2.2101, we detected a high lipolytic activity by utilizing myo-inositol and glycerol under oxic conditions. Furthermore, a constitutive expression of a phospholipase enzyme was measured for this species. Myo-inositol and glycerol is thought to derive from the degradation of meat cells, as described by Love and Pearson (1971). Under anoxic conditions, *B. thermosphacta* TMW2.2101 is predicted to utilize the meat derived metabolite ethanolamine yielding energy-rich acetyl-CoA. This is in accordance to previous studies describing *B. thermosphacta* as major player in fatty meat products (Grau, 1983; Talon et al., 1992). Carbon sources utilized under oxic conditions by *C. divergens* TMW2.1577, comprise the amino acids glutamine as well as glycerol and allantoin, whereas under anoxic conditions purine and pyrimidine bases are metabolized. Enzymes for allantoin degradation could not be found in the genomes of *B. thermosphacta* and both *Leuconostoc* species. In addition to ribose, *L. gelidum* subsp. *gasicomitatum* predictively prefers pentoses as carbon source, indicated by an upregulation of several enzymes of the pentose phosphate pathway under anoxic conditions.

This study contributes to a better understanding of previous research which have been done concerning microbial coexistence on meat regarding nutrition utilization and adaptation to MAs. It further evaluates the potential of the industrial used protective gases for red and white meat packaging in order to prevent meat spoilage by the species analyzed.

## Conclusion

Protective gases such as oxygen and carbon dioxide are used in food industry to prevent bacterial cell growth and spoilage on meat. Nevertheless, we demonstrated that different spoilers encode for different metabolic pathways to cope with the detrimental effects of oxygen and carbon dioxide on their metabolism. By controlled regulation of those metabolic pathways, bacteria developed strategies to cope with high oxygen amounts, e.g., consumption of oxygen, reduction of oxidative stress or regulation of oxygen sensitive enzymes as well as high amounts of carbon dioxide, e.g., maintenance of intracellular pH, maintenance of osmotic balance and adaptation of the cell membrane by altering the fatty acid composition. Thus, the usage of MAs only prevents bacterial spoilage to a certain degree.

Furthermore, it appears that high oxygen concentrations (70%_O_2_) antagonize with high CO_2_ concentrations (30%_CO_2_), i.e., counteracting the desired inhibitory effect of carbon dioxide on bacteria upon MAP. This effect could be demonstrated in this study for *B. thermosphacta* and *C. divergens*, which are inhibited by CO_2_ only in the absence of high oxygen concentrations. No inhibition by CO_2_ was demonstrated for both *L. gelidum* subspecies and *C. maltaromaticum* enabling their growth on meat independent of the MA applied. Proteomic data also demonstrated that different spoilers utilize distinct organic substrates available on meat explaining their coexistence as a microbial spoilage consortium on meat.

## Data Availability Statement

All LC-MS/MS data files and MaxQuant output files have been deposited to the ProteomeXchange Consortium (http://proteomecentral.proteomexchange.org) via the PRIDE partner repository with the dataset identifier PXD016382.

## Author Contributions

SK designed the study, performed the experiments and data evaluation, and wrote the first draft of the manuscript. CL performed the mass spectrometric data acquisition. CM supervised the data evaluation. MH helped to draft the manuscript, assisted in data interpretation, and supervised the work of SK. RV initiated the project and supervised the work of SK. All authors read and approved the final manuscript.

## Conflict of Interest

The authors declare that the research was conducted in the absence of any commercial or financial relationships that could be construed as a potential conflict of interest.
