# Supplementary material for: Comparative Proteomics of Meat Spoilage Bacteria Predicts Drivers for Their Coexistence on Modified Atmosphere Packaged Meat
Source: Front Microbiol. 2020 Feb 14;11:209. doi: 10.3389/fmicb.2020.00209 (PMC7033586; doi:10.3389/fmicb.2020.00209)
Supplement: Supplementary file 1 [file Data_Sheet_1.docx]

Supplementary Material

# Supplementary figures

**Figure S1** Cell growth of the five meat-spoiling bacteria *L. gelidum* subsp. *gelidum* TMW2.1618, *L. gelidum* subsp. *gasicomitatum* TMW2.1619, *C. divergens* TMW2.1577, *C. maltaromaticum* TMW2.1581 and *B. thermosphacta* TMW2.2101 under the four gas atmospheres (◇) air, (☐) N_2_, (▲) 30%_CO_2_/70%_N_2_, (■) 30%_CO_2_/70%_O_2_. Yellow marked dots represent the time point of proteomic sample taking.

# Supplementary tables

**Table S1** Perseus output of all proteins of each species after statistical data analysis based on LFQ values. A Welch´s t-test was performed to identify statistically significant regulated proteins between two gas atmospheres. Furthermore, iBAQ values for all protein of each species are given after data cleanup. Table S1 is provided as a separate Excel file.

**Table S2**

Total number of proteins encoded in the genome according to the NCBI annotation, identified by MaxQuant search after clean-up of raw data and significantly differentially (p<0.05, log_2_ fold change >=2) up- (⇧) or down- (⇩) regulated under each sampling condition for the meat-spoilage bacteria *B. thermosphacta* TMW2.2101, *C. divergens* TMW2.1577, *C. maltaromaticum* TMW2.1581, *L. gelidum* subsp. *gelidum* TMW2.1618 and *L. gelidum* subsp. *gasicomitatum* TMW2.1619.

|  | Encoded | Detected | Air_vs_N_2_ | | Air_vs_CO_2__O_2_ | | | Air_vs_CO_2__N_2_ | | N_2__vs_CO_2__N_2_ | | | CO_2__O_2__vs_CO_2__N_2_ | |
| --- | --- | --- | --- | --- | --- | --- | --- | --- | --- | --- | --- | --- | --- | --- |
|  |  |  | ⇧ | ⇩ | ⇧ | ⇩ | ⇧ | | ⇩ | ⇧ | ⇩ | ⇧ | | ⇩ |
| *B. thermosphacta* TMW2.2101 | 2285 | 1696 | 34 | 20 | 2 | 1 | 44 | | 26 | 0 | 21 | 47 | | 33 |
| *C. divergens* TMW2.1577 | 2490 | 1811 | 25 | 29 | 0 | 0 | 19 | | 8 | 0 | 1 | 33 | | 41 |
| *C. maltaromaticum* TMW2.1581 | 3205 | 2152 | 39 | 16 | 26 | 32 | 16 | | 21 | 4 | 44 | 46 | | 40 |
| *L. gelidum* subsp. *gelidum* TMW2.1618 | 1605 | 1375 | 5 | 4 | 14 | 2 | 4 | | 1 | 0 | 0 | 3 | | 14 |
| *L. gelidum* subsp. *gasicomitatum* TMW2.1619 | 1740 | 1420 | 14 | 9 | 15 | 10 | 26 | | 15 | 5 | 1 | 16 | | 9 |
